# Computational Aspects of N-Mixture Models

**DOI:** 10.1111/biom.12246

**Published:** 2014-10-14

**Authors:** Emily B Dennis, Byron JT Morgan, Martin S Ridout

**Affiliations:** 1 School of Mathematics, Statistics and Actuarial Science, University of KentCanterbury, Kent CT2 7NF, UK

**Keywords:** Abundance estimation, Method of moments, Multivariate negative binomial, Multivariate Poisson, Optimal design, Sampling, Temporal replication

## Abstract

The N-mixture model is widely used to estimate the abundance of a population in the presence of unknown detection probability from only a set of counts subject to spatial and temporal replication (Royle, 2004, *Biometrics* **60**, 105–115). We explain and exploit the equivalence of N-mixture and multivariate Poisson and negative-binomial models, which provides powerful new approaches for fitting these models. We show that particularly when detection probability and the number of sampling occasions are small, infinite estimates of abundance can arise. We propose a sample covariance as a diagnostic for this event, and demonstrate its good performance in the Poisson case. Infinite estimates may be missed in practice, due to numerical optimization procedures terminating at arbitrarily large values. It is shown that the use of a bound, *K*, for an infinite summation in the N-mixture likelihood can result in underestimation of abundance, so that default values of *K* in computer packages should be avoided. Instead we propose a simple automatic way to choose *K*. The methods are illustrated by analysis of data on Hermann's tortoise *Testudo hermanni*.

## 1. Introduction

Estimating the abundance of a population is an important component of ecological research. N-mixture models can be used to estimate animal abundance from counts with both spatial and temporal replication whilst accounting for imperfect detection (Royle, 2004a). Whereas alternative sampling methods for obtaining estimates of abundance exist, such as capture–recapture, distance, removal and multiple-observer sampling, these may be expensive in effort or cost, or impractical for some species and scenarios. A benefit of the N-mixture model is the reasonably low comparative cost and effort required for data collection which does not require individuals to be identified. This is especially true of many citizen-science based monitoring programs.

Consequently, since development by Royle (2004a), many applications and extensions of the N-mixture model have been made. These include applications to various taxa, including birds (Kéry, Royle, and Schmid, 2005), mammals (Zellweger-Fischer, Kéry, and Pasinelli, 2011), and amphibians (Dodd and Dorazio, 2004; McIntyre et al., 2012). In addition, covariates have often been used to examine spatial patterns in abundance and detection (Kéry, 2008) and hence create maps of spatial abundance (Royle, Nichols, and Kéry, 2005).

Despite the popularity of the N-mixture model, few studies have made comparisons with estimates derived via alternative methods or undertaken simulation studies of performance (Kéry et al., 2005; Hunt, Weckerly, and Ott, 2012; Couturier et al., 2013). A potential issue for fitting the model using classical inference is the need to specify an upper bound, *K*, to approximate an infinite summation in the likelihood. We found this matter was rarely mentioned in publications. For example, McIntyre et al. (2012) used simulated data to support their amphibian study, highlighting the benefit of more sampling occasions, particularly when detection probability was low, however the value of *K* used was not provided. When software such as unmarked (Fiske and Chandler, 2011) written in R (R Core Team, 2014) and PRESENCE (Hines, 2011) is used for model fitting, it is possible that only default values of the bound are employed. Couturier et al. (2013) suggest bias could be induced by the choice of *K* for low detection probabilities.

In this article, we investigate computational aspects of fitting N-mixture models, in particular via a simulation study for scenarios where detection probability is low and/or the number of sampling occasions is small. This may be important for the study of cryptic species, and have implications for sample design: many applications to date have made only three visits, whereas in Royle (2004a) simulations were tested for five visits and an application made to data with 10 visits. When only one sampling visit is made, it is well known that the N-mixture model reduces to a thinned Poisson distribution, with only one estimable parameter, the product of mean abundance and detection probability, a feature which underlies aspects of the work which follows.

The N-mixture model is described in Section The N-Mixture Model. In Section Equivalence of the Poisson N-Mixture Model With a Multivariate Poisson Model we explain the equivalence of the Poisson N-mixture model with a multivariate Poisson distribution. We use this formulation to show that infinite estimates of abundance may arise, and provide a simple diagnostic to identify such cases. The multivariate Poisson formulation has the advantage of not requiring a constant *K* to be set. Section Explicit Form for the Bivariate Negative-Binomial Case provides the probability function in the bivariate negative-binomial case. In Section The Effect of the Choice of *K* on Fitting the N-Mixture Model: Poisson Case, we show how the choice of *K* in the N-mixture model interacts with the occurrence of infinite estimates of abundance, and how incorrect conclusions may arise. An automatic method for choosing *K* is provided. Section Moment Estimation for a Mixed-Poisson N-Mixture Model provides moment estimates and evaluates the use of two diagnostic tests for the negative-binomial case for when infinite estimates of abundance may arise. Section Application to Hermann's Tortoise Data provides an application to real data and the article ends with discussion and recommendations.

## 2. The N-Mixture Model

Under the study design in Royle (2004a), a set of counts is made during sampling visits 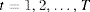 at 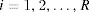 locations (sites). The population is assumed to be closed during the period of sampling and each individual is assumed to have the same detection probability *p*. The counts 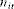 at site *i* and time *t* are assumed to be independent binomial random variables,


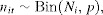


where 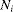 is the unknown population size at site *i*. To fit the model using classical inference, we assume the 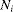 to be independent random variables with probability function 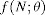, and then maximize the likelihood


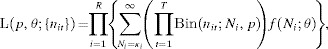
(1)

where 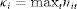. As noted by Royle (2004a), numerical maximization of 2013 requires the replacement of the infinite summation over 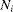 by a sum with upper limit *K*. The value of *K* may be selected by fitting the model for a succession of increasing values and selecting *K* when the parameter estimates appear to stabilize (Royle, 2004a). We shall consider both Poisson and negative-binomial mixing distributions.

It is our experience that the N-mixture model can produce unrealistically large estimates of abundance and we explain this feature in the article.

## 3. Equivalence of the Poisson N-Mixture Model With a Multivariate Poisson Model

The number of individuals observed at a site at time *t* can be written as the convolution of independent random variables, corresponding to those seen only once, those seen twice, etc. This natural feature of the N-mixture model can be formalized as we now show.

Let 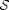 denote the set of non-empty subsets of 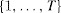, and let the random variable 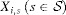 denote the number of individuals seen at site *i* only on occasion *s*. For example, 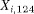 denotes the individuals seen at site *i* on occasions 1, 2, and 4 only. Then, if we let 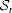 denote those elements of 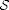 that include *t*, we can decompose 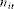 as


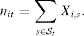


For example, with 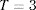, we have


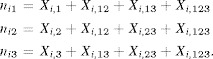


Conditional on 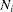, the joint distribution of the set of random variables 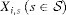 is multinomial, with index 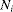 and probabilities 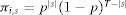, where 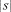 denotes the number of elements in the set *s*. When 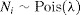, the 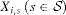 are independent Poisson random variables, with


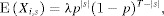


see Johnson, Kotz, and Balakrishnan (1997, p. 146). The thinned Poisson is the case 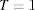.

It follows that the joint distribution of 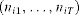 is multivariate Poisson (Johnson et al., 1997, Chapter 37), with


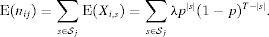


There are 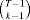 subsets 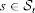 such that 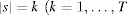). Hence


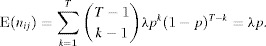


Similarly, if we let 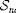 denote the elements of 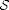 that include both *t* and *u* then


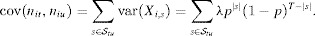


There are 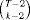 subsets 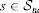 such that 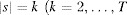). Hence, for 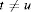,


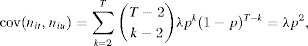


and 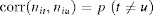.

This result is a special case of Johnson et al. (1997, equation 37.88), which is stated without proof.

### Example: T=2, Poisson Case

Cormack (1989) mentions this case in closed-population capture–recapture modeling of data from one site only.

Suppressing site dependence, we have


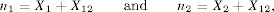


where 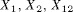 are independent with 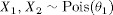, where 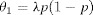 and 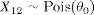, where 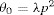. Note that small *p* would result typically in small values for 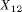, and as *p* tends to zero 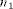 and 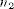 become independent, so that the model reverts to a thinned Poisson.

The counts 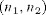 follow a bivariate Poisson distribution with 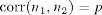, and the bivariate Poisson probability is


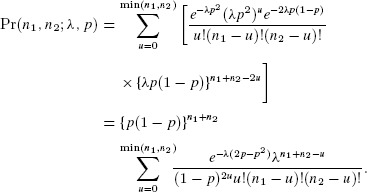
(2)

Including site dependence, the likelihood is


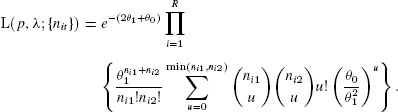
(3)

For 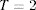 the expressions of 2013 and 2011 are identical, but the likelihood of 2011 may be maximized without requiring selection of a value *K*.

### 3.1. Multivariate Poisson Distribution

For general *T*, let 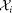 denote the set of all possible values 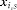 of the random variables 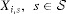 such that


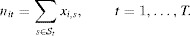


Because the random variables 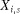 are independent, the joint probability function of 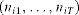 is


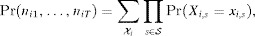


and


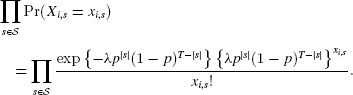


There are 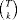 elements 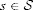 such that 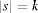, for 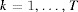. Hence


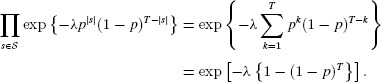


Therefore, we can write


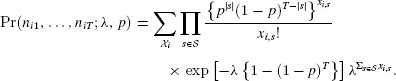
(4)

The case 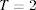 is given in 2011. The associated R program incorporates efficient construction of 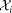.

### 3.2. Performance of the Multivariate Poisson Model

**Figure 1:**
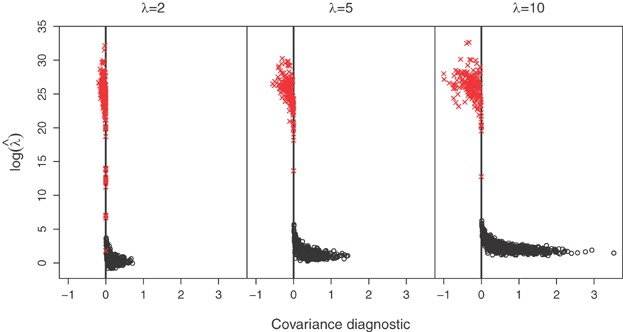
Log(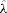) from the bivariate Poisson model plotted against the covariance diagnostic, cov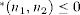 from 2008, based upon 1000 simulated datasets for 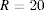, 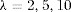 and 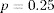. Values at which the covariance diagnostic is negative are shown by crosses. This figure appears in color in the electronic version of this article.

For illustration, we investigate performance of the multivariate Poisson model via simulation from the fitted model. We assess output for the cases 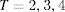 based upon 1000 simulations where 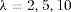, 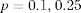 and 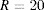. The chosen parameter values were guided by those used in Royle (2004a). The model was fitted using the optim function in the R software package (R Core Team, 2014) using the default Nelder–Mead algorithm and a tolerance value of 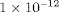. The results were checked with those from using several other optim algorithms, including simulated annealing and quasi-Newton. We observe that estimates for 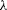 were very large in some cases (the maximum estimate from 1000 simulations was 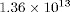 when 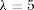, 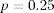, and 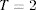). Figure1 shows that non-positive values of a covariance diagnostic,


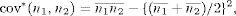
(5)

can identify the high estimates of 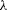 from fitting the bivariate Poisson. Here 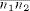 denotes the mean of the product 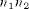 over *S* sites. Note that this (intraclass) estimate is appropriate as 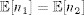. A proof that a local maximum of the likelihood occurs at 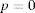 when 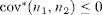 is given in the Appendix; we are working on a general proof for 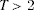, as well as a proof that there are no other maxima when the diagnostic is satisfied. Hence, in these instances when 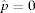, in order to have finite 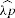, 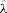 is actually infinite and the large range of high estimates of abundance obtained in practice, as in Figure1, is partly an artefact of the optimization routine stopping prematurely when the likelihood is flat.

For more than two visits (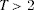), the appropriate covariance diagnostic can be estimated as


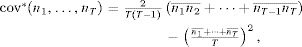
(6)

where the first term consists of the average of the means of all 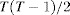 pairwise products. Our conjecture that the diagnostic extends for 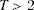 is supported by Web Figure 4 which compares the covariance diagnostic 2009 with 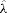 from the multivariate Poisson model for 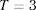, when 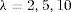.

Performance of the covariance diagnostic is demonstrated further in Table 1, which shows close correspondence between the proportion of simulations where the diagnostic is negative and the proportion where 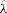 is large (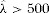). Table 1 also shows the prevalence of infinite estimates of 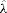, particularly as 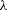, *T*, and *p* decrease. In fact for the case where 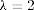, 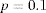, and 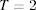, a finite value of 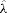 was not achievable in over half of 1000 simulations.

**Table 1:** Performance of the covariance diagnostic for the multivariate Poisson model, based upon 1000 simulations for various scenarios of 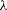, *p*, and *T* for 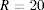 sites. EPN is the proportion of simulations when the sample covariance diagnostic was negative. EPD is the proportion of simulations where the estimate of 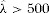

		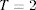		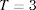		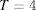	
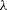	*p*	EPN	EPD	EPN	EPD	EPN	EPD
2	0.10	0.505	0.505	0.351	0.351	0.276	0.276
2	0.25	0.225	0.224	0.090	0.089	0.033	0.033
5	0.10	0.427	0.427	0.362	0.361	0.219	0.222
5	0.25	0.167	0.167	0.084	0.084	0.017	0.020
10	0.10	0.398	0.398	0.317	0.318	0.251	0.256
10	0.25	0.180	0.181	0.066	0.066	0.038	0.038

## 4. Explicit Form for the Bivariate Negative-Binomial Case

The Poisson distribution may be replaced by a mixed-Poisson distribution, for which 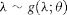, when the probability of 2011 becomes


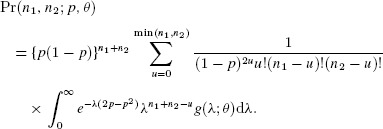


For the negative-binomial distribution, the mixing distribution is gamma with parameters 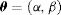 and


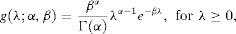
(7)

which results in the NB-2 form (Hilbe, 2011, p. 187). In this case


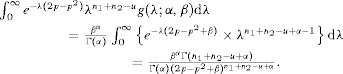


Therefore the joint probability for the bivariate negative-binomial model is given by


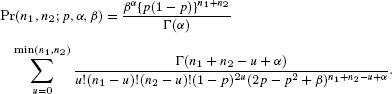
(8)

In the parameterization of 2011, the mean and variance of the gamma distribution are 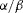 and 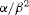, respectively. If we now write 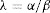 for the expected value of the Poisson mean, then the variance is 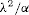 and the coefficient of variation of the Poisson mean is 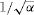. The Poisson model arises as the limit 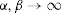, maintaining 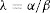.

In terms of the parameters 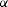 and 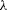, 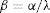 and we can write 2014 as


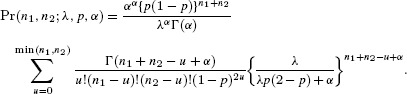
(9)

The case for 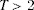 follows in the same way, by integrating the expression of 2003, to give the multivariate negative-binomial probability as


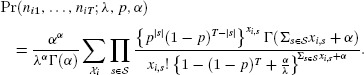


The expression 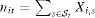 also applies to the negative binomial case, but the 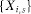 are no longer independent.

## 5. The Effect of the Choice of *K* on Fitting the N-Mixture Model: Poisson Case

### 5.1. Incorrect Estimates due to the Choice of *K*

We now consider how the choice of *K* for computing the Poisson N-mixture likelihood of 2013 interacts with the occurrence of infinite estimates of 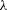. Output is obtained for 1000 simulations based on the parameter values used in Royle (2004a), where 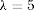, 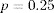 and 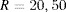, but for number of sampling occasions 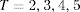. The models were again fitted using optim in the R software package. The parameters *p* and 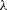 were constrained to be in range via logit and log link functions, respectively. Each simulated dataset was fitted with 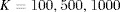.

**Figure 2:**
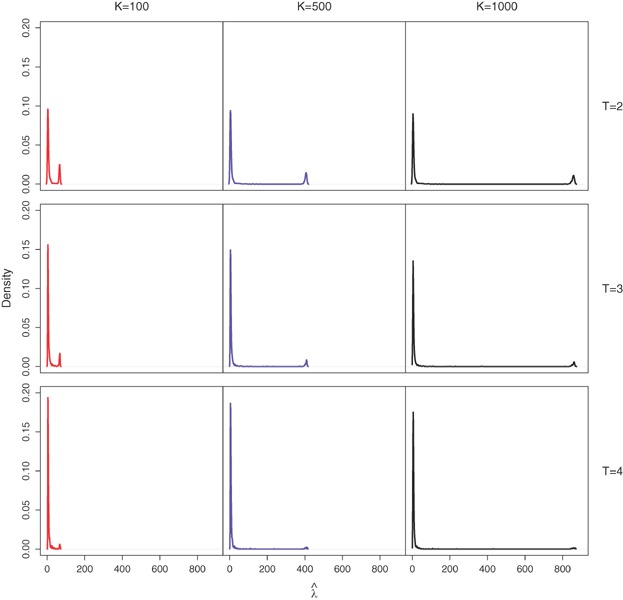
Kernel density estimates of 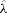 from the Poisson N-mixture model for 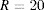 sites, 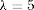 and 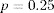 based upon 1000 simulated datasets for 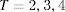, and 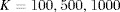. This figure appears in color in the electronic version of this article.

We see that large finite estimates of abundance can arise, in particular where the number of sampling occasions *T* is small (Figure2). Specifically, a proportion of simulations result in a second peak in the sampling distribution for 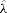 and the value at which this is found increases with the value of *K*. Fitting the multivariate Poisson model to simulated data created under comparable scenarios for 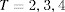 also produced a second peak in the sampling distribution for 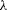, but as described in Section Performance of the Multivariate Poisson Model, the estimates were substantially greater in the absence of the limiting value *K* in the N-mixture model. An increase in the number of sampling occasions reduces the incidence of high estimates of 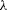, which become rare for 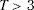, as more information is available as *T* increases. For 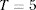 very few high estimates of 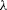 occurred in the 1000 simulations. An increase in the number of sites also reduces the proportion of high values (Web Figure 5).

Thus when the N-mixture model is fitted by maximizing the likelihood of 2013, when 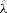 should be infinite, 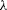 is estimated as large as possible for a given value of *K*, and 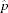 is restricted to be as close to zero as possible. We discuss this matter further in Web Appendix 1. The occurrence of large finite estimates of 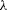 is similar to analogous findings of Wang and Lindsay (2005) in the context of species richness estimation.

### 5.2. Automatic Choice of *K*

For the Poisson case the covariance diagnostic identifies when infinite values of 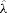 arise. When the diagnostic is not satisfied, *K* may be selected automatically, for example by ensuring that the Poisson upper tail probability is 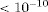, so that the value of *K* will adapt for successive iterations according to the estimate of 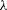. This approach was also suggested by Guillera-Arroita et al. (2012). We have found this to be a simple and preferable alternative to fitting the model for successively larger values of *K* until estimates appear to stabilize.

## 6. Moment Estimation for a Mixed-Poisson N-Mixture Model

Suppose we have an N-mixture model in which 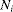 follows a mixed-Poisson distribution, as in Section Explicit Form for the Bivariate Negative-Binomial Case, with


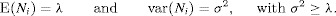


Conditional on 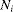, the random variables 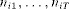 are independent binomial variables, with


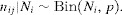


Therefore, conditional on 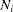



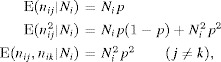


and the corresponding unconditional expectations are


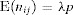
(10)


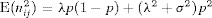
(11)


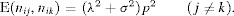
(12)

It follows that


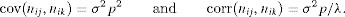


### 6.1. Moment Estimation

We have the following moment estimates for 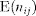, 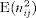, and 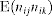, respectively:


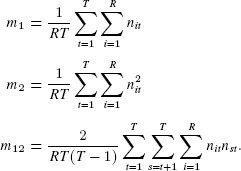


Equating these to the expectations given by (10)–(12) yields the following moment estimators of the parameters 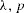, and 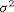



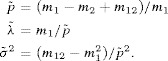


Because 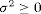, we require


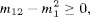
(13)

for a valid set of moment estimates. This is the same diagnostic as used previously in 2009.

We also require 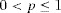. The lower bound yields the new diagnostic


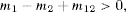
(14)

for a finite (moment) estimate of 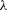. The upper bound yields


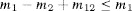


or


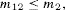


which is a consequence of the Cauchy–Schwarz inequality and not a useful diagnostic. The bound 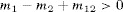 given above to ensure 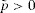 and hence 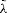 finite, gives a new diagnostic.

If we adopt a method-of-moments (MOM) approach for the bivariate Poisson distribution, *p* is estimated by the sample correlation of the counts, as observed also by Royle (2004b), and 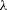 is estimated by dividing 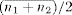 by this estimate of *p*. For more than two visits (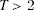), *p* can be estimated by the mean of all sample correlations between counts for different sampling occasions. Then 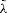 is the sample mean of all counts divided by this estimate of *p*. This generalizes Holgate's (1964) work, which considered 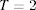 only. In Web Appendix 2 we assess the performance of MOM estimation as a simple method for parameter estimation compared to maximum likelihood for the N-mixture model.

### 6.2. Performance of the Multivariate Negative-Binomial Model

**Figure 3:**
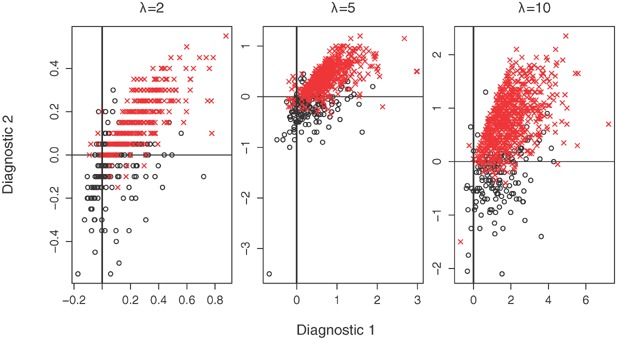
Diagnostic 1 (13) versus diagnostic 2 (14) from the bivariate negative binomial model, based upon 1000 simulated datasets for 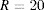, 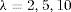, 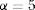, and 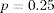. Values at which 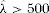 and 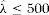 are shown by circles and crosses, respectively. This figure appears in color in the electronic version of this article.

Given the proposed diagnostics for the mixed-Poisson case in Section Moment Estimation, here we assess the performance of the multivariate negative-binomial model. Simulated data were fitted as in Section Performance of the Multivariate Poisson Model but for the negative binomial case, with 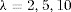 and 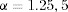. We again assume that 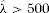 equates to infinite 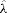. If both (13)and (14) are negative, 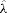 is very likely to be infinite and the mean proportion with 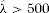 from 21 scenarios is 0.921 (Table 2). However performance of the diagnostics when one or more of the two diagnostics is negative is less clear. Additionally, 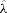 may occasionally be infinite despite both diagnostics being positive and on average 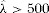 for approximately 8.5% of simulations when both diagnostics are positive. Performance for the bivariate cases where 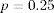 and 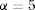 is illustrated in Figure 3 and for the cases where 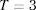 and 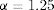 in Web Figures 6–8. We see that neither singly nor in combination do the diagnostics perform as well as the single diagnostic for the Poisson case. We see fewer instances of infinite 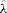 for large *T* and *p*.

**Table 2:** Performance of the covariance diagnostic for the multivariate negative-binomial model, based upon 1000 simulations for various scenarios of 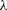, *p*, 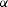, and *T* for 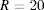 sites. EP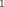, EP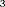, and EP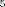 are the proportion of simulations where both diagnostics are negative, one or more diagnostic is negative, or both diagnostics are positive, respectively. EP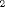, EP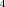, and EP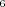 are the corresponding proportions of those where 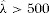

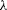	*p*	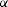	*T*	EP 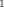	EP 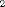	EP 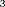	EP 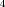	EP 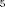	EP 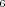
2	0.10	1.25	2	0.192	0.938	0.3	0.853	0.388	0.072
2	0.10	1.25	3	0.093	0.925	0.271	0.841	0.426	0.131
2	0.10	5.00	2	0.199	0.92	0.296	0.804	0.274	0.113
2	0.10	5.00	3	0.104	0.904	0.264	0.822	0.293	0.126
2	0.25	1.25	2	0.046	0.913	0.229	0.777	0.571	0.07
2	0.25	1.25	3	0.002	1	0.138	0.681	0.71	0.048
2	0.25	5.00	2	0.064	0.953	0.184	0.826	0.411	0.097
2	0.25	5.00	3	0.011	1	0.103	0.748	0.473	0.047
5	0.10	1.25	2	0.088	0.966	0.347	0.813	0.472	0.121
5	0.10	1.25	3	0.023	1	0.333	0.757	0.52	0.113
5	0.10	5.00	2	0.139	0.935	0.305	0.803	0.282	0.128
5	0.10	5.00	3	0.064	0.906	0.252	0.829	0.343	0.143
5	0.25	1.25	2	0.006	1	0.217	0.71	0.746	0.068
5	0.25	1.25	3	0	–	0.137	0.533	0.843	0.047
5	0.25	5.00	2	0.038	0.763	0.193	0.741	0.555	0.05
5	0.25	5.00	3	0.002	0.5	0.108	0.694	0.678	0.028
10	0.10	1.25	2	0.032	0.969	0.342	0.813	0.596	0.139
10	0.10	1.25	3	0.005	1	0.325	0.775	0.65	0.097
10	0.10	5.00	2	0.116	0.931	0.322	0.835	0.378	0.108
10	0.10	5.00	3	0.027	0.926	0.302	0.844	0.437	0.105
10	0.25	1.25	2	0	–	0.193	0.674	0.806	0.069
10	0.25	1.25	3	0	–	0.125	0.472	0.87	0.029
10	0.25	5.00	2	0.01	0.9	0.156	0.756	0.726	0.054
10	0.25	5.00	3	0.001	1	0.09	0.656	0.817	0.026

## 7. Application to Hermann's Tortoise Data

Here we analyze data from a study of the threatened Hermann's tortoise *Testudo hermanni* in southeastern France. One hundred and eighteen sites were each surveyed three times during a period when the species is most active. Full details are provided in Couturier et al. (2013), and we briefly reassess the conclusions drawn in their article and demonstrate the effect of study design on results.

For the tortoise data, optimization of the negative-binomial model confirms that 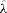 is infinite in the negative binomial model for these data; after 500 iterations, the estimates had reached


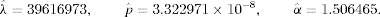


As noted in Couturier et al. (2013), the fit is much improved compared to the Poisson case, with -maximum log-likelihood 540.34 versus 576.27, but at the expense of 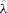 becoming infinite. Hence for this dataset a finite estimate of mean abundance can be obtained for the Poisson but not for the negative-binomial. Whilst the first diagnostic (13) is positive, 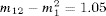, so that the Poisson estimate is finite, the additional diagnostic (14) is negative, 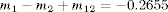.

The zero-inflated Poisson is an intermediate model between the Poisson and negative-binomial, with -maximum log-likelihood 562.13 for these data. The zero-inflated Poisson therefore provides an improvement upon the Poisson case, but still yields the finite parameter estimate 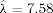.

To show the potential effect of study design on model performance, we inspect the sample covariance diagnostic (13) for this dataset for the Poisson case for a reduced number of sites and/or visits. Taking two of the three visits made at all sites, the diagnostic was always positive (0.97–1.17). The diagnostic based upon all three visits but a random sample of fewer sites, was negative for 1.7% and 0% of 1000 samples, respectively for 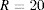 and 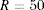. However for only two visits, the diagnostic was negative for 9.0% and 0.8% of 1000 samples, respectively for 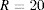 and 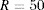.

## 8. Discussion and Recommendations

We have shown that the N-mixture model can produce infinite estimates of abundance, particularly when working with a limited number of sampling occasions and low detection probability. The equivalence of the N-mixture model with the multivariate Poisson has been demonstrated, allowing us to understand and diagnose poor behavior of the N-mixture model.

We believe the equivalence of the Poisson N-mixture model to the multivariate Poisson distribution to be previously largely unknown, especially in statistical ecology. The multivariate Poisson model conveniently avoids the requirement to select an upper bound *K*. We provide code for fitting the multivariate Poisson and negative-binomial models. Possible alternative techniques for fitting the multivariate distributions include using the EM algorithm (Karlis, 2003), a composite likelihood (Jost, Brcich, and Zoubir, 2006) or a symbolic computation approach (Sontag and Zeilberger, 2010). Consequently this equivalence could also have the alternative purpose of using the N-mixture model to provide simple fitting of the multivariate Poisson and negative-binomial models for particular covariance structures.

A recent extension of the N-mixture model to open populations by including population dynamics parameters offers great potential but also requires an upper bound to be set (Dail and Madsen, 2011). Further exploration of this model via simulation to assess performance is in progress. Kéry et al. (2009) extended the N-mixture model to allow for analysis of data resulting from closed sampling periods connected by open periods and the multivariate formulations also apply in that case. Dorazio, Martin, and Edwards (2013) provide an extension in which *p* is given a distribution at each visit. The binomial distribution in 2013 is then replaced by a beta-binomial. This has also been considered in a Bayesian context by Martin et al. (2011). For the multivariate Poisson case this extension is dealt with by appropriate numerical integration of the probability of 2003. An increasing number of studies use a Bayesian approach for parameter estimation (Kéry et al., 2009; Graves et al., 2011). Further simulation study comparing a Bayesian approach with maximum-likelihood estimation could show whether this approach can also produce poor estimates in some scenarios. Some comparisons have been made by Toribio, Gray, and Liang (2012), based upon parameter values from Royle (2004a).

In practice, covariates are frequently used to describe variation in abundance and detection. Further analysis could determine how the inclusion of covariates might affect instances where a finite abundance estimate cannot be obtained for a model with constant abundance and detection, and in particular determine whether parameters may become identifiable.

Good experimental design is vital for occupancy studies; see for example Guillera-Arroita, Ridout, and Morgan, (2010, 2014). The same issues apply for N-mixture work, though with the different perspective of avoiding poor model-fitting behavior. If possible, study design effort should be distributed to ensure more than two visits are made to each site (in addition to including a reasonable number of sites). Alternatively a study design where more visits are made to a subset of the sites is worth exploring.

For maximum-likelihood estimation, we recommend using MOM estimates to start the iterative search for MLEs. In the Poisson case the covariance diagnostic may be used to determine when infinite estimates of abundance may arise. Infinite estimates of abundance may occur for some model choices but not others, as for the Hermann's Tortoise case study. Hence we advise fitting the model for multiple distribution choices, to identify which may provide finite estimates of abundance. An R program is available in the Supplementary Materials which allows for covariates in the detection and abundance parameters.

## 9. Supplementary Materials

The Web Appendices referenced in Sections Performance of the Multivariate Poisson Model, Incorrect Estimates due to the Choice of *K*, and Performance of the Multivariate Negative-Binomial Model, together with R code, are available with this paper at the *Biometrics* website on Wiley Online Library.

## Appendix

### Appendix

*Proof that when*  *a local maximum of the likelihood occurs at*  *when cov*.

It is convenient here to set . It is simple to prove that , as noted in Holgate (1964). For observation , we write

The profile log-likelihood function for  is then given by

We deduce that

Thus

and
