# Supplementary material for: Computational Aspects of N-Mixture Models
Source: Biometrics. 2014 Oct 14;71(1):237–46. doi: 10.1111/biom.12246 (PMC4406156; doi:10.1111/biom.12246)
Supplement: Supplementary file 1 [file biom0071-0237-sd1.pdf]

Web-based Supplementary Materials for

Computational aspects of N-mixture models

by Emily B. Dennis, Byron J.T. Morgan & Martin S. Ridout

Web Appendix 1

The effect of the choice of K on fitting the N-mixture model

Section 5 of the paper demonstrates that the infinite values of $\hat{\lambda}$ shown in Section 3.2 for the bivariate Poisson are limited by the value of K adopted in the corresponding Poisson N-mixture model. Here this effect of K is discussed further.

Web Figure 1a illustrates the effect of K for a single simulated dataset, with $\hat{\lambda}$ increasing linearly with K. The corresponding relationships from different simulations and parameter values are found to be very similar. The heuristic reason for this, and the fact that the green line in Web Figure 1a lies below the line of unit slope through the origin, is that in the mixture in (1), for large λ the Poisson distribution is approximately Normal, N(λ,λ). For values of K in the approximate range $\lambda \pm 2\sqrt{\lambda}$, the effect of K is to lose a large fraction of this probability, and hence reduce the likelihood. Therefore it would not be possible to estimate λ values that correspond to reduced values of the likelihood and thus in practice $K > \hat{\lambda}$.

For a negative-binomial mixing distribution, we find that where $\hat{\lambda}$ is infinite for the Poisson distribution, the green solid line in Web Figure 1a is unchanged for the negative binomial. However, for a different simulation (blue lines), when the sample covariance diagnostic (5) is positive, for a Poisson mixing distribution, $\hat{\lambda} = 6.64$ for increasing K, but using the negative binomial $\hat{\lambda}$ increases with K but with a smaller slope than that of the Poisson line. Hence the single covariance diagnostic is not sufficient for the negative-binomial distribution, which can

produce large estimates that increase with K in cases where the diagnostic is positive and $\hat{\lambda}$ from the Poisson distribution is stable. We encounter this latter case again in Section 7. An additional diagnostic for the negative-binomial case is given in Section 6 and explored via simulation in Web Appendix 2.

Web Figure 1b shows that as the value of $\hat{\lambda}$ increases, the smaller eigenvalue of the Hessian matrix of the log likelihood evaluated at the maximim-likelihood estimate, estimated within `optim`, decreases towards zero. The model becomes near singular (Catchpole et al., 2001), with only the product λp being estimable, corresponding to the thinned Poisson situation. Estimates from K=200 and K=1000 are equivalent for finite $\hat{\lambda}$ (and hence overlap in the main peak in Figure 2), but differ when $\hat{\lambda}$ should be infinite and $\hat{\lambda}$ approaches K. In particular, the spread of non-zero eigenvalues when $\hat{\lambda}$ is close to K is reduced for larger K (Web Figure 1b). The artificial truncation of the range of λ by K (demonstrated in Figure 2 and Web Figure 5) is responsible for the non-zero values of the smaller eigenvalue for the largest values of $\hat{\lambda}$ (Web Figure 1b).

The sampling distribution of the product $\widehat{\lambda p}$ is unbiased (Web Figure 2), hence when finite estimation of $\hat{\lambda}$ is impossible, only a single thinned-Poisson parameter λp is estimable, the hyperbola for which is shown in Web Figure 3a. Web Figure 3b illustrates the log-log transform of Web Figure 3a, rotated 135° about the axis to examine possible differences for an increasing number of visits, T. The main distribution shows similar spread for different values of T but fewer small estimates of λ as more visits are made. For cases where $\hat{\lambda}$ is truncated by K, estimates of λ do not vary with T, as found also when the green line of Web Figure 1a did not vary for alternative parameter values. However corresponding estimates of p (not given here) show less variation for greater T, corresponding to the greater information available for more visits.

References

Catchpole, E. A., Kgosi, P. M., and Morgan, B. J. T. (2001). On the near-singularity of models for animal recovery data. *Biometrics* **57,** 720–726.

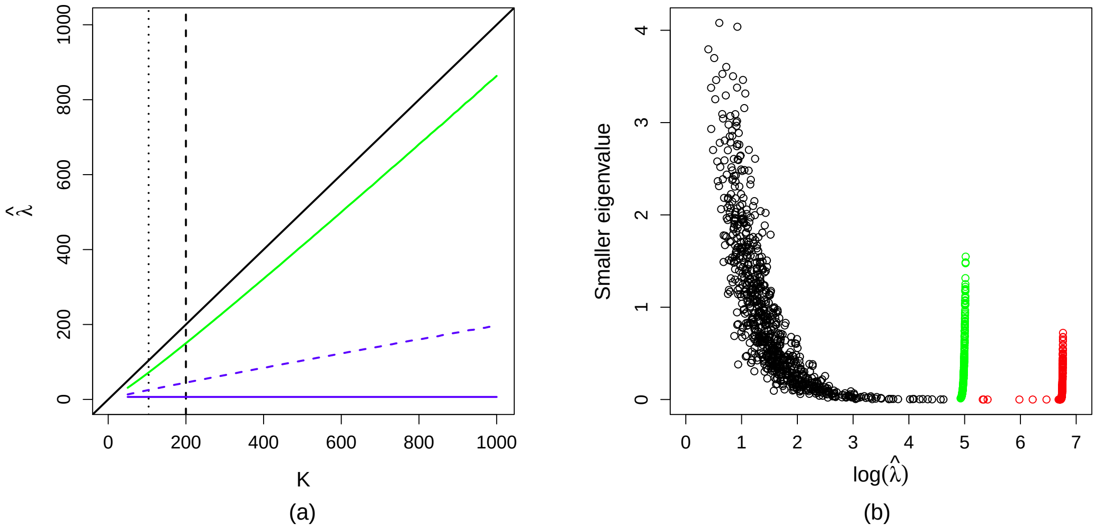

Web Figure 1: a) $\hat{\lambda}$ plotted against increasing K for a single simulation (green) with default values of K for `unmarked` (K = max(count) + 100, dotted) and PRESENCE ($K = 200$, dashed) also shown. $\hat{\lambda}$ is plotted against increasing K for a different simulation in blue, with a comparison of λ estimates for a Poisson (solid) and negative-binomial (dashed) mixing distribution. b) A plot of $\log(\hat{\lambda})$ versus the smaller eigenvalue of the estimated Hessian at the maximum-likelihood estimate for $K = 200$ & 1000 (black), $K = 200$ (green) and $K = 1000$ (red) based upon 1000 simulated datasets. The parameter values used were $T = 2$, $R = 20$, $\lambda = 5$ and $p = 0.25$.

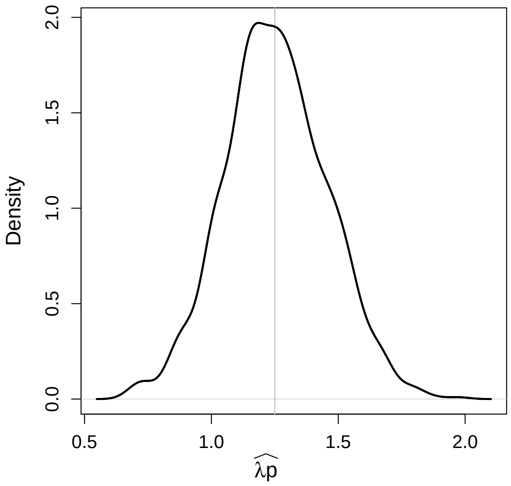

Web Figure 2: Kernel density estimate for $\widehat{\lambda p}$ from the Poisson N-mixture model, based upon 1000 simulated datasets for $K = 200$, $T = 2$ and $R = 20$, $\lambda = 5$ and $p = 0.25$.

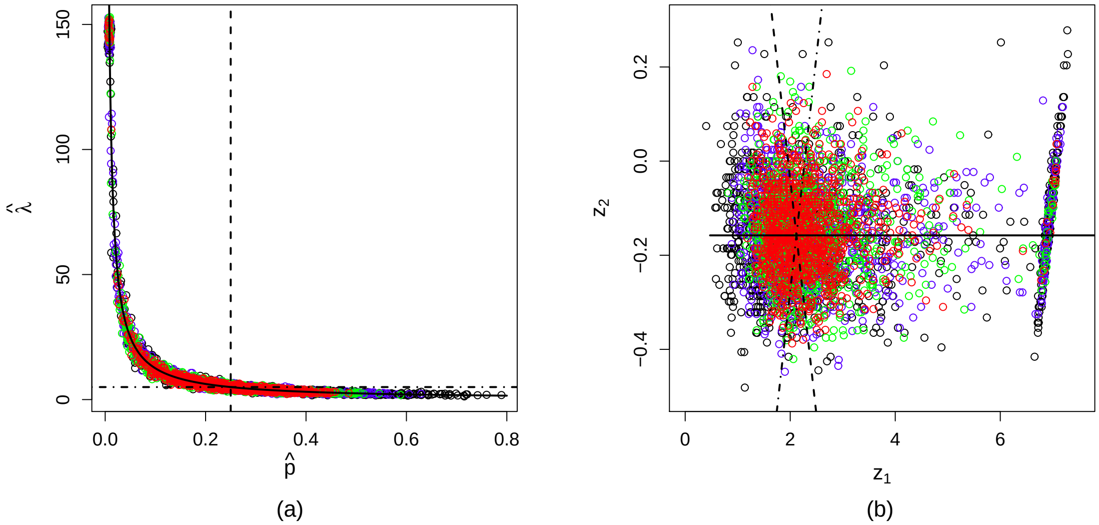

Web Figure 3: (a) A plot of $\hat{\lambda}$ versus $\hat{p}$ and (b) $\log(\hat{\lambda})$ versus $\log(\hat{p})$ rotated 135° clockwise about the origin, for $T = 2$ (black), $T = 3$ (blue), $T = 4$ (green) and $T = 5$ (red) based upon 1000 simulated datasets for $K = 200$, $R = 20$, $\lambda = 5$ and $p = 0.25$. $z_1 = -\frac{1}{\sqrt{2}}\log\hat{p} + \frac{1}{\sqrt{2}}\log\hat{\lambda}$ and $z_2 = -\frac{1}{\sqrt{2}}\log\hat{p} - \frac{1}{\sqrt{2}}\log\hat{\lambda}$. In a) the solid line represents the hyperbola for λp and the straight lines correspond to the known values of p (dashed) and λ (dot-dash). In b) the straight lines represent the rotated logarithms of λp (solid), p (dashed) and λ (dot-dash).

Web Appendix 2

Performance of the method-of-moments estimation

We assess the performance of MOM estimation as a simple method for parameter estimation compared to maximum likelihood estimation (MLE) from the N-mixture model. For the Poisson case, estimates for where the covariance diagnostic is negative were excluded in this comparison. Correspondingly, estimates for the negative-binomial were excluded when one or more of the diagnostics was negative. Additionally, for both the Poisson and negative-binomial, cases where either the MLE or MOM estimate of λ is finite but large ($\hat{\lambda} > 100$) were excluded to provide a fair comparison.

For the Poisson case, when $p = 0.25$, the MOM approach only performs better than MLE based upon RMSE when $T = 2$ (Web Table 1). However for smaller $p = 0.10$, MOM estimation performs better for almost all cases (Web Table 2). In the negative-binomial case, results are not greatly affected by varying α (Web Tables 3-6). As in the Poisson case, when $p = 0.25$, MOM only outperforms MLE when few visits are made, which is emphasised when λ is small. For smaller $p = 0.10$, MOM often performed better than MLE in terms of RMSE, although the difference was reduced for increasing T and λ.

Method of moments can quickly provide good estimates of λ and p, but it does not consistently outperform MLE. We suggest using MOM estimates as sensible starting values for optimisation of the N-mixture likelihood.

Web Table 1: Comparison of estimation via method-of-moments (MOM) and the N-mixture model (MLE) for the Poisson case with $\lambda = 2, 5, 10$, $p = 0.25$ and $R = 20$ for 1000 simulations. RMSE is the root mean-squared error for λ. The upper bound K for the MLE was automatically selected such that the tail proportion was 10^{-10}. EPD is the proportion of simulations discarded when the covariance diagnostic was negative or either estimate of $\hat{\lambda} > 100$. EPN is the proportion of simulations when both diagnostics were negative.

a) $\lambda = 2$

T	Method	Mean	Median	RMSE	EPD	EPN
2	MLE	2.28	1.58	2.49	0.312	0.219
	MOM	1.9	1.41	1.5		
3	MLE	2.53	1.89	2.66	0.18	0.098
	MOM	2.43	1.63	2.61		
4	MLE	2.73	2.01	3.19	0.091	0.033
	MOM	2.97	1.82	4.03		
5	MLE	2.7	2.04	3.11	0.055	0.012
	MOM	2.93	1.93	4.37		

b) $\lambda = 5$

T	Method	Mean	Median	RMSE	EPD	EPN
2	MLE	5.97	3.87	7.92	0.258	0.171
	MOM	5.45	3.64	5.72		
3	MLE	6.95	4.74	7.97	0.152	0.083
	MOM	6.68	4.27	9.01		
4	MLE	6.44	4.93	6.74	0.081	0.017
	MOM	6.97	4.57	8.29		
5	MLE	6.47	5.04	6.03	0.046	0.008
	MOM	6.59	4.8	6.63		

c) $\lambda = 10$

T	Method	Mean	Median	RMSE	EPD	EPN
2	MLE	11.5	8.07	11.52	0.255	0.157
	MOM	9.96	7.21	8.79		
3	MLE	12.89	9.51	11.72	0.147	0.072
	MOM	12.39	8.76	11.82		
4	MLE	12.89	9.87	10.72	0.094	0.029
	MOM	12.78	9.34	11.44		
5	MLE	12.14	10.08	7.82	0.055	0.016
	MOM	12.42	9.53	9.87		

Web Table 2: Comparison of estimation via method-of-moments (MOM) and the N-mixture model (MLE) for the Poisson case with $\lambda = 2, 5, 10$, $p = 0.1$ and $R = 20$ for 1000 simulations. RMSE is the root mean-squared error for λ. The upper bound K for the MLE was automatically selected such that the tail proportion was 10^{-10}. EPD is the proportion of simulations discarded when the covariance diagnostic was negative or either estimate of $\hat{\lambda} > 100$. EPN is the proportion of simulations when both diagnostics were negative.

a) $\lambda = 2$

T	Method	Mean	Median	RMSE	EPD	EPN
2	MLE	1.1	0.77	1.51	0.61	0.52
	MOM	0.71	0.61	1.36		
3	MLE	1.87	1.12	2.41	0.506	0.377
	MOM	1.25	0.94	1.22		
4	MLE	2.43	1.4	3.4	0.444	0.283
	MOM	1.58	0.98	1.51		
5	MLE	2.65	1.58	3.97	0.356	0.214
	MOM	1.94	1.21	2.27		

b) $\lambda = 5$

T	Method	Mean	Median	RMSE	EPD	EPN
2	MLE	3.19	1.97	5.62	0.52	0.424
	MOM	2.25	1.67	3.31		
3	MLE	4.6	2.84	5.79	0.467	0.331
	MOM	3.53	2.18	4.09		
4	MLE	6.33	3.63	9.04	0.387	0.235
	MOM	5.42	2.7	7.45		
5	MLE	6.71	4.08	8.64	0.364	0.203
	MOM	5.39	3.09	7.92		

c) $\lambda = 10$

T	Method	Mean	Median	RMSE	EPD	EPN
2	MLE	6.72	4.01	8.8	0.527	0.451
	MOM	4.73	3.17	6.82		
3	MLE	10.16	6	12.5	0.433	0.299
	MOM	8.21	4.55	11		
4	MLE	12.5	7.96	13.99	0.387	0.223
	MOM	9.72	5.85	11.73		
5	MLE	11.59	7.69	11.78	0.384	0.174
	MOM	10.53	6.76	12.59		

Web Table 3: Comparison of estimation via method-of-moments (MOM) and the N-mixture model (MLE) for the negative-binomial case with $\lambda = 2, 5, 10$, $p = 0.25$, $\alpha = 1.25$, and $R = 20$ for 1000 simulations. RMSE is the root mean-squared error for λ. The upper bound K for the MLE was automatically selected such that the tail proportion was 10^{-10}. EPD is the proportion of simulations discarded when either covariance diagnostic was negative or either estimate of $\hat{\lambda} > 100$. EPN is the proportion of simulations when both diagnostics were negative.

a) $\lambda = 2$

T	Method	Mean	Median	RMSE	EPD	EPN
2	MLE	2.24	1.44	4.22	0.256	0.058
	MOM	1.81	1.3	1.67		
3	MLE	2.52	1.72	3.52	0.185	0.013
	MOM	2.49	1.57	3.33		
4	MLE	2.64	1.78	3.6	0.129	0
	MOM	2.84	1.71	5.37		
5	MLE	2.7	1.84	4.1	0.093	0
	MOM	2.89	1.79	5.31		

b) $\lambda = 5$

T	Method	Mean	Median	RMSE	EPD	EPN
2	MLE	5.42	3.44	8.58	0.258	0.009
	MOM	5.2	3.16	7.67		
3	MLE	6.43	4.35	8.13	0.192	0
	MOM	6.14	3.93	7.8		
4	MLE	5.99	4.32	5.82	0.151	0
	MOM	6.82	4.34	8.25		
5	MLE	5.88	4.56	5.49	0.1	0
	MOM	6.63	4.52	7.56		

c) $\lambda = 10$

T	Method	Mean	Median	RMSE	EPD	EPN
2	MLE	10.66	7.07	11.3	0.287	0.001
	MOM	9.32	6.36	9.11		
3	MLE	11.49	8.55	9.29	0.207	0
	MOM	11.74	7.92	12.1		
4	MLE	11.76	8.79	10.52	0.135	0
	MOM	11.45	8.38	10.24		
5	MLE	11.68	9.12	9.08	0.106	0
	MOM	11.44	8.4	10.2		

Web Table 4: Comparison of estimation via method-of-moments (MOM) and the N-mixture model (MLE) for the negative-binomial case with $\lambda = 2, 5, 10$, $p = 0.1$, $\alpha = 1.25$, and $R = 20$ for 1000 simulations. RMSE is the root mean-squared error for λ. The upper bound K for the MLE was automatically selected such that the tail proportion was 10^{-10}. EPD is the proportion of simulations discarded when either covariance diagnostic was negative or either estimate of $\hat{\lambda} > 100$. EPN is the proportion of simulations when both diagnostics were negative.

a) $\lambda = 2$

T	Method	Mean	Median	RMSE	EPD	EPN
2	MLE	1.02	0.64	1.92	0.565	0.401
	MOM	0.67	0.51	1.4		
3	MLE	1.48	0.91	2.38	0.45	0.187
	MOM	1.1	0.8	1.35		
4	MLE	1.7	1.06	2.48	0.425	0.097
	MOM	1.36	0.9	1.55		
5	MLE	2.58	1.38	6.25	0.349	0.044
	MOM	1.7	1.08	1.92		

b) $\lambda = 5$

T	Method	Mean	Median	RMSE	EPD	EPN
2	MLE	2.69	1.68	5.13	0.455	0.161
	MOM	1.96	1.41	3.51		
3	MLE	3.74	2.29	5.51	0.405	0.048
	MOM	3.32	2	4.51		
4	MLE	5.19	2.73	8.69	0.364	0.012
	MOM	4.47	2.36	6.91		
5	MLE	5.46	2.95	8.93	0.348	0.002
	MOM	4.53	2.67	7.88		

c) $\lambda = 10$

T	Method	Mean	Median	RMSE	EPD	EPN
2	MLE	5.79	3.36	9.68	0.439	0.037
	MOM	4.28	2.89	7.02		
3	MLE	7.63	4.24	11.08	0.373	0.002
	MOM	6.13	3.94	7.75		
4	MLE	8.84	5.47	10.57	0.373	0
	MOM	8.64	5.1	11.67		
5	MLE	8.73	5.7	10.27	0.35	0
	MOM	8.3	5.14	9.72		

Web Table 5: Comparison of estimation via method-of-moments (MOM) and the N-mixture model (MLE) for the negative-binomial case with $\lambda = 2, 5, 10$, $p = 0.25$, $\alpha = 5$, and $R = 20$ for 1000 simulations. RMSE is the root mean-squared error for λ. The upper bound K for the MLE was automatically selected such that the tail proportion was 10^{-10}. EPD is the proportion of simulations discarded when either covariance diagnostic was negative or either estimate of $\hat{\lambda} > 100$. EPN is the proportion of simulations when both diagnostics were negative.

a) $\lambda = 2$

T	Method	Mean	Median	RMSE	EPD	EPN
2	MLE	2.38	1.6	2.88	0.301	0.151
	MOM	1.79	1.32	1.57		
3	MLE	2.62	1.88	3.1	0.195	0.052
	MOM	2.33	1.67	2.36		
4	MLE	2.91	1.95	4.74	0.098	0.011
	MOM	2.9	1.91	4.05		
5	MLE	2.5	1.98	2.49	0.052	0.003
	MOM	2.85	1.86	4.28		

b) $\lambda = 5$

T	Method	Mean	Median	RMSE	EPD	EPN
2	MLE	6.25	3.89	7.86	0.243	0.065
	MOM	5.6	3.6	7.07		
3	MLE	6.51	4.47	7.77	0.129	0.01
	MOM	6.41	4.13	8.62		
4	MLE	6.6	4.86	6.48	0.094	0.001
	MOM	6.93	4.67	8.21		
5	MLE	6.58	4.88	7.44	0.07	0.001
	MOM	6.92	4.64	8.15		

c) $\lambda = 10$

T	Method	Mean	Median	RMSE	EPD	EPN
2	MLE	11.11	7.55	10.41	0.235	0.02
	MOM	10.45	7	10.94		
3	MLE	12.08	8.8	11.23	0.153	0
	MOM	12.52	8.57	12.35		
4	MLE	12.34	9.45	10.68	0.091	0
	MOM	12.29	9.01	10.74		
5	MLE	11.84	9.51	8.03	0.053	0
	MOM	12.44	9.33	10.08		

Web Table 6: Comparison of estimation via method-of-moments (MOM) and the N-mixture model (MLE) for the negative-binomial case with $\lambda = 2, 5, 10$, $p = 0.1$, $\alpha = 5$, and $R = 20$ for 1000 simulations. RMSE is the root mean-squared error for λ. The upper bound K for the MLE was automatically selected such that the tail proportion was 10^{-10}. EPD is the proportion of simulations discarded when either covariance diagnostic was negative or either estimate of $\hat{\lambda} > 100$. EPN is the proportion of simulations when both diagnostics were negative.

a) $\lambda = 2$

T	Method	Mean	Median	RMSE	EPD	EPN
2	MLE	1	0.65	1.44	0.605	0.497
	MOM	0.7	0.6	1.4		
3	MLE	1.56	1.04	2.15	0.482	0.291
	MOM	1.18	0.82	1.28		
4	MLE	2.25	1.31	3.45	0.454	0.187
	MOM	1.6	1.06	1.62		
5	MLE	2.75	1.56	5.47	0.393	0.139
	MOM	1.81	1.17	2.05		

b) $\lambda = 5$

T	Method	Mean	Median	RMSE	EPD	EPN
2	MLE	3.39	1.86	6.29	0.517	0.301
	MOM	2.19	1.56	3.32		
3	MLE	4.73	2.61	7.9	0.449	0.174
	MOM	3.45	2.18	4.17		
4	MLE	5.4	3.34	8.1	0.384	0.092
	MOM	4.36	2.73	5.7		
5	MLE	5.82	3.64	7.9	0.342	0.063
	MOM	5.66	2.99	10.01		

c) $\lambda = 10$

T	Method	Mean	Median	RMSE	EPD	EPN
2	MLE	7.09	4.13	9.52	0.466	0.171
	MOM	5.51	3.61	6.99		
3	MLE	8.42	5.19	10	0.393	0.075
	MOM	7.34	4.55	9.28		
4	MLE	9.72	5.96	10.9	0.36	0.045
	MOM	9.13	5.55	10.7		
5	MLE	9.76	6.65	10.56	0.343	0.015
	MOM	9.94	5.85	11.41		

Web Appendix 3

Supplementary tables and figures

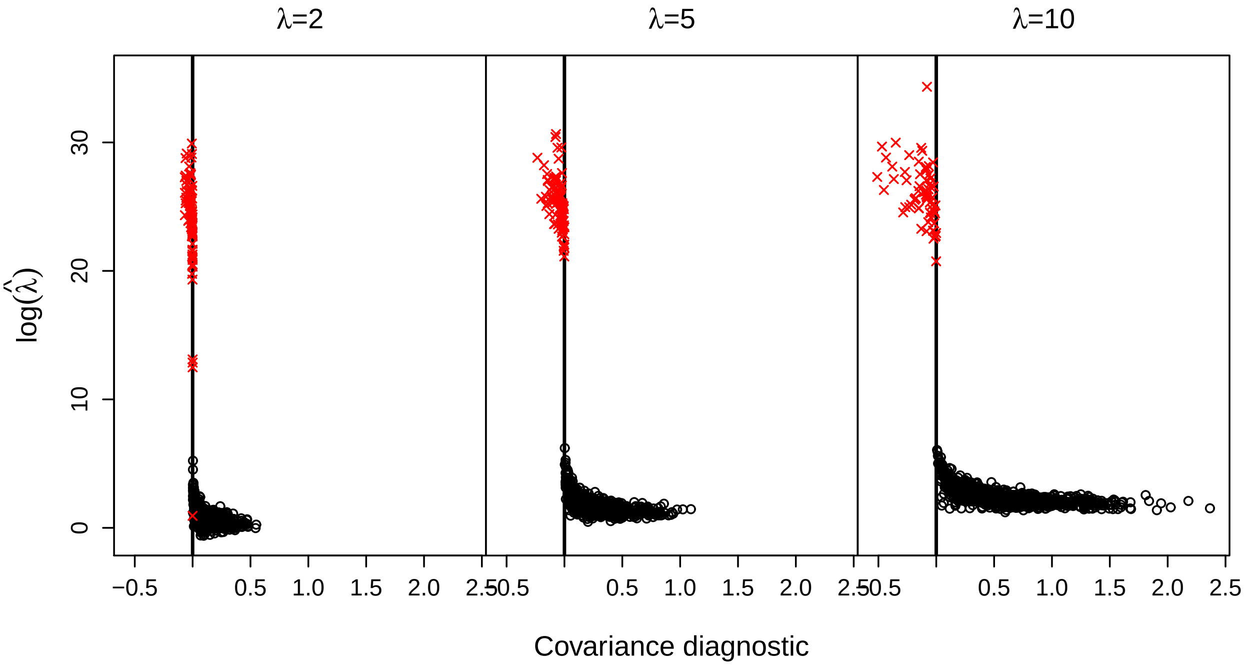

Web Figure 4: Log($\hat{\lambda}$) from the multivariate Poisson model with $T = 3$ plotted against the covariance diagnostic, $\text{cov}^*(n_1, n_2, n_3) \leq 0$ from (6), based upon 1000 simulated datasets for $R = 20$, $\lambda = 2, 5, 10$ and $p = 0.25$. Values at which the covariance diagnostic is negative are shown by crosses.

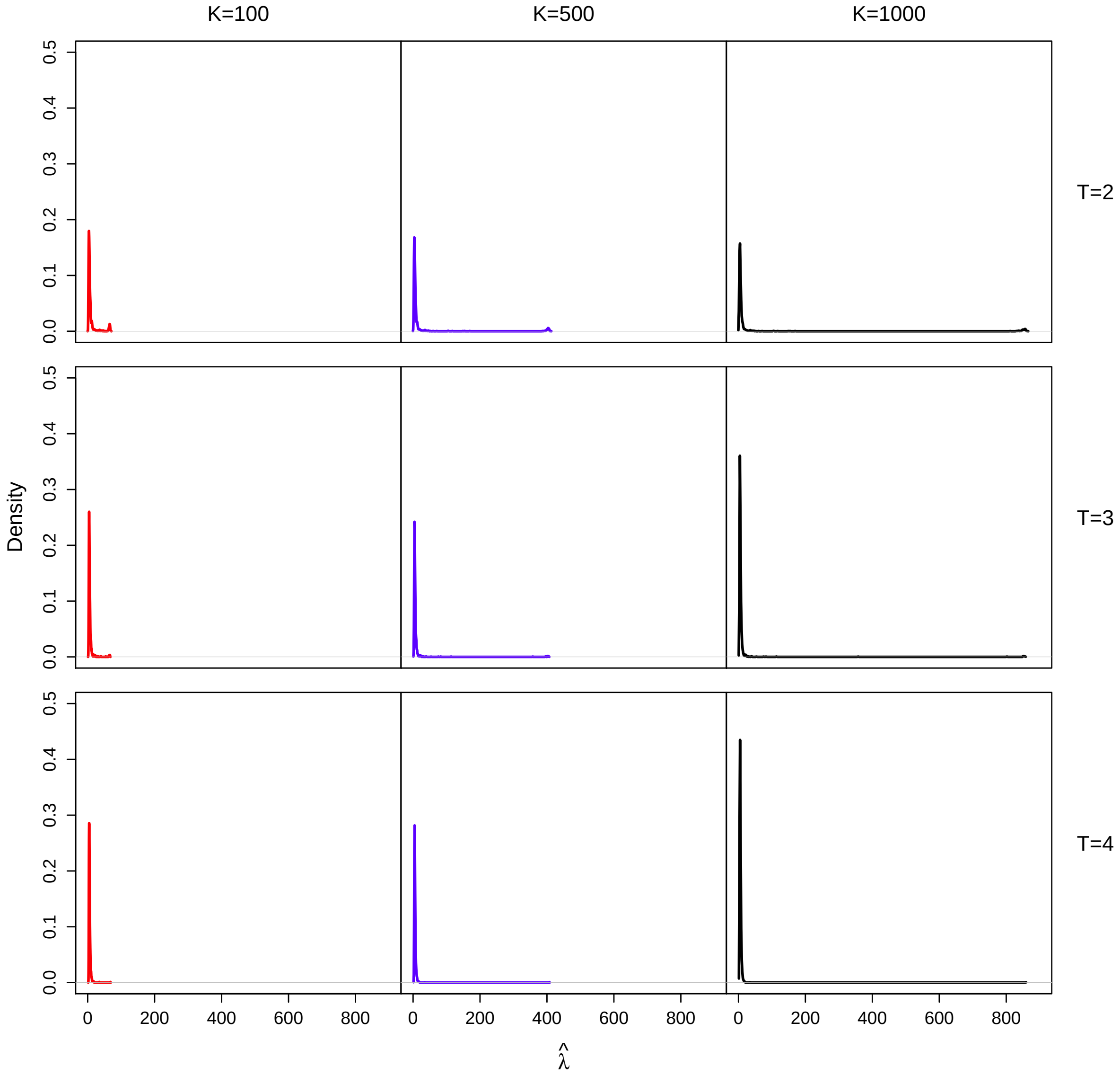

Web Figure 5: Kernel density estimates of $\hat{\lambda}$ from the Poisson N-mixture model for $R = 50$ sites, $\lambda = 5$ and $p = 0.25$ based upon 1000 simulated datasets for $T = 2, 3, 4$ and $K = 100$ (red), 500 (blue) and 1000 (black).

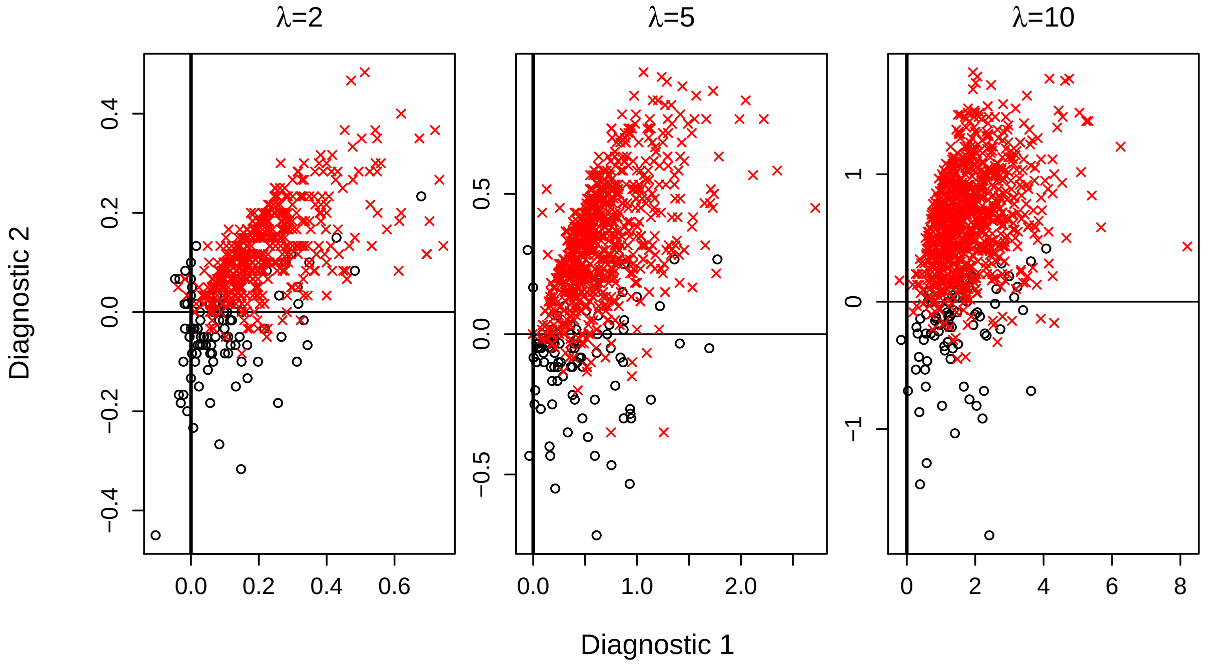

Web Figure 6: Diagnostic 1 (13) versus diagnostic 2 (14) from the multivariate negative binomial model when $T = 3$, based upon 1000 simulated datasets for $R = 20$, $\lambda = 2, 5, 10$, $\alpha = 5$ and $p = 0.25$. Values where $\hat{\lambda} > 500$ and $\hat{\lambda} \leq 500$ are shown by circles and crosses, respectively.

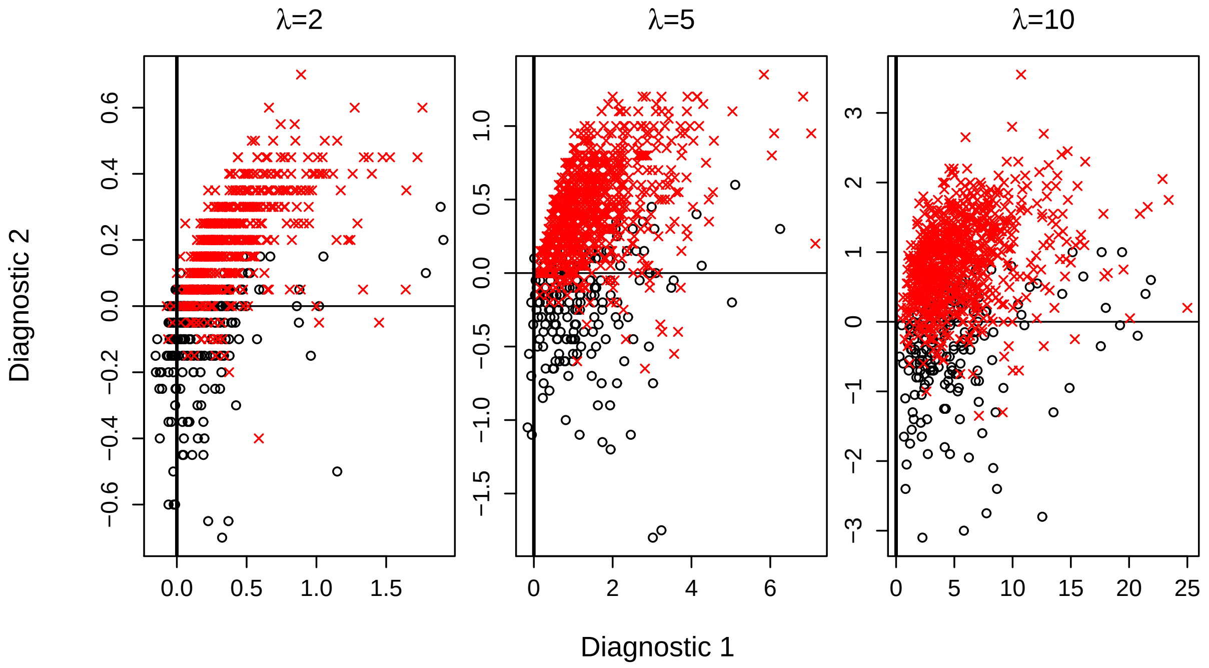

Web Figure 7: Diagnostic 1 (13) versus diagnostic 2 (14) from the bivariate negative binomial model, based upon 1000 simulated datasets for $R = 20$, $\lambda = 2, 5, 10$, $\alpha = 1.25$ and $p = 0.25$. Values where $\hat{\lambda} > 500$ and $\hat{\lambda} \leq 500$ are shown by circles and crosses, respectively.

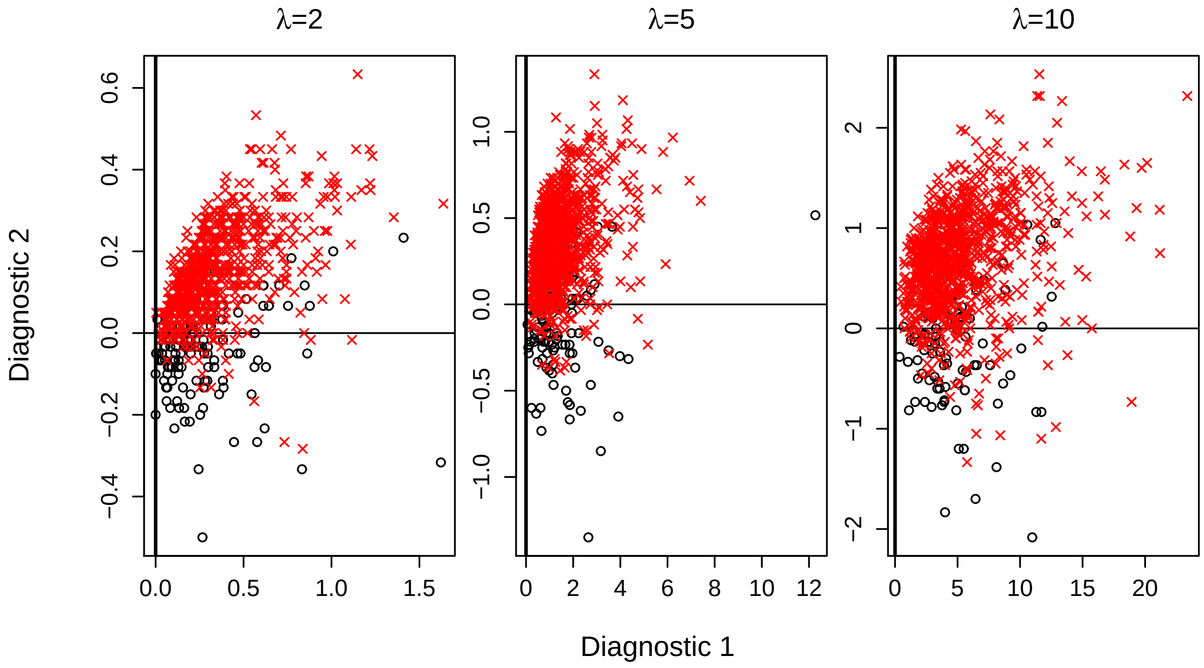

Web Figure 8: Diagnostic 1 (13) versus diagnostic 2 (14) from the multivariate negative binomial model when $T = 3$, based upon 1000 simulated datasets for $R = 20$, $\lambda = 2, 5, 10$, $\alpha = 1.25$ and $p = 0.25$. Values where $\hat{\lambda} > 500$ and $\hat{\lambda} \leq 500$ are shown by circles and crosses, respectively.
